# Bioassay-Guided Fractionation of *Melastoma malabathricum* Linn. Leaf Solid Phase Extraction Fraction and Its Anticoagulant Activity

**DOI:** 10.3390/molecules20033697

**Published:** 2015-02-24

**Authors:** Li Teng Khoo, Janna Ong Abdullah, Faridah Abas, Eusni Rahayu Mohd Tohit, Muhajir Hamid

**Affiliations:** 1 Department of Microbiology, Faculty of Biotechnology and Biomolecular Sciences, Universiti Putra Malaysia, 43400 Serdang, Selangor, Malaysia; E-Mails: litengkhoo@gmail.com (L.T.K.); janna@upm.edu.my (J.O.A.); 2 Department of Food Science, Faculty of Food Science and Technology, Universiti Putra Malaysia, 43400 Serdang, Selangor, Malaysia; E-Mail: faridah_abas@upm.edu.my; 3 Institute of Biosciences, Universiti Putra Malaysia, 43400 Serdang, Selangor, Malaysia; 4 Department of Pathology, Faculty of Medicine and Health Sciences, Universiti Putra Malaysia, 43400 Serdang, Selangor, Malaysia; E-Mail: eusni@upm.edu.my

**Keywords:** bioassay-guided fractionation, *Melastoma malabathricum*, anticoagulant, cinnamic acid, cinnamic acid derivative, mixing study

## Abstract

The aims of this study were to examine the bioactive component(s) responsible for the anticoagulant activity of *M. malabathricum* Linn. leaf hot water crude extract via bioassay-guided fractionation and to evaluate the effect of bioactive component(s) on the intrinsic blood coagulation pathway. The active anticoagulant fraction of F3 was subjected to a series of chromatographic separation and spectroscopic analyses. Furthermore, the effect of the bioactive component(s) on the intrinsic blood coagulation pathway was studied through immediate and time incubation mixing studies. Through Activated Partial Thromboplastin Time (APTT) assay-guided fractionation, Subfraction B was considered the most potent anticoagulant fraction. Characterisation of Subfraction B indicated that anticoagulant activity could partly be due to the presence of cinnamic acid and a cinnamic acid derivative. APTT assays for both the immediate and time incubation mixing were corrected back into normal clotting time range (35.4–56.3 s). In conclusion, cinnamic acid and cinnamic acid derivative from Subfraction **B** were the first such compounds to be discovered from *M. malabathricum* Linn. leaf hot water crude extract that possess anticoagulant activity. This active anticoagulant Subfraction **B** prolonged blood clotting time by causing factor(s) deficiency in the intrinsic blood coagulation pathway.

## 1. Introduction

The secondary haemostasis defence mechanism of blood coagulation is a cascade reaction for limiting proteolysis of various coagulant factors in the active form [1,2]. Through a series of zymogen proteolytic activations in the coagulation pathway, the final key enzyme, thrombin, converts the soluble fibrinogen into the insoluble fibrin for formation of blood clots. It acts together with aggregated platelets at the site of injury to prevent blood loss [3]. However, alteration of blood clotting factors could promote development of high thrombosis morbidity and mortality [4,5]. Henceforth, conventional anticoagulants are used widely in treating and preventing thrombosis, but they contribute to bleeding and other life threatening side effects [6,7,8]. For examples, unfractionated heparin and warfarin caused heparin-induced thrombocytopenia (HIT) and drug-drug or drug-food interaction, respectively [9,10]. Frequent monitoring and dose adjustments for these anticoagulants with patients are needed [11]. Besides, no antagonist to neutralise the adverse effects of newly developed F Xa and thrombin inhibitors [12,13]. Therefore, research in finding alternative anticoagulants especially from medicinal plants with similar efficacy as conventional, and newly developed anticoagulants with minimal side effects are highly sought after [14,15,16].

*Melastoma malabathricum* Linn. is a traditional perennial medicinal herb that grows abundantly in tropical and subtropical countries [17,18]. Previous study showed that *M. malabathricum* Linn. leaf was effective in prolonging the blood clotting time through the intrinsic pathway [19]. Recently, we also demonstrated that fractions from *M. malabathricum* Linn. leaf hot water crude extract significantly prolonged the blood clotting times in a dose dependent manner. Anticoagulant activities of the fractions were suggested to be due to the presence of acidic polysaccharides and polyphenolics [20]. However, till now there is no updated research regarding active anticoagulant component(s) from *M. malabathricum* Linn. leaf. Triterpenes and flavonoids isolated from *M. malabathricum* Linn. leaf were claimed to play major roles in its antioxidant, anticancer, antiplatelet and other pharmacological activities [21,22,23]. Hence, in this study, it was hypothesised that *M. malabathricum* Linn. hot water crude leaf extract also contained bioactive component(s) responsible for its anticoagulant activity. As a continuation of recent findings, further examinations were conducted to identify the active components with anticoagulant activities. The active anticoagulant fraction of *M. malabathricum* Linn. leaf was subjected to bioassay guided fractionation. Activated partial thromboplastin time (APTT), a functional-clot assay, was employed as a guide for measurement of the blood clotting activities for each separated fraction. Structural identification and elucidation for the active anticoagulant compounds were also performed using high performance liquid chromatography (HPLC), mass spectroscopy (MS) and nuclear magnetic resonance (NMR). The nature of the single active fraction together with the activity of identified chemical components on the intrinsic blood coagulation pathway was also evaluated.

## 2. Results and Discussion

**Figure 1:**
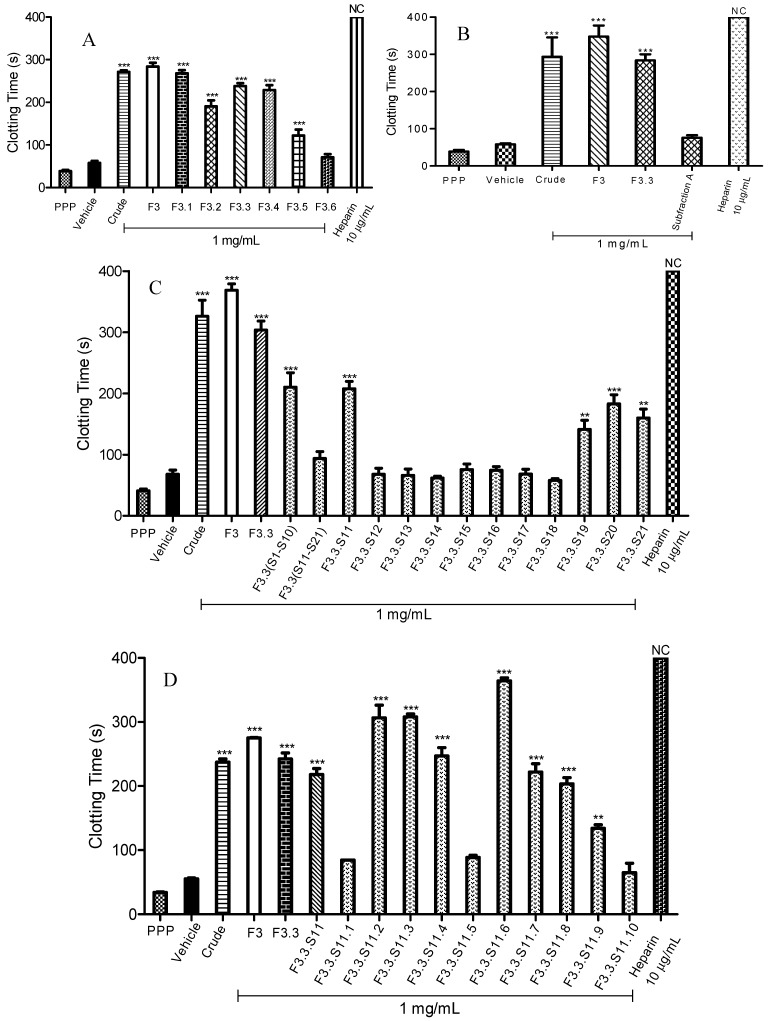

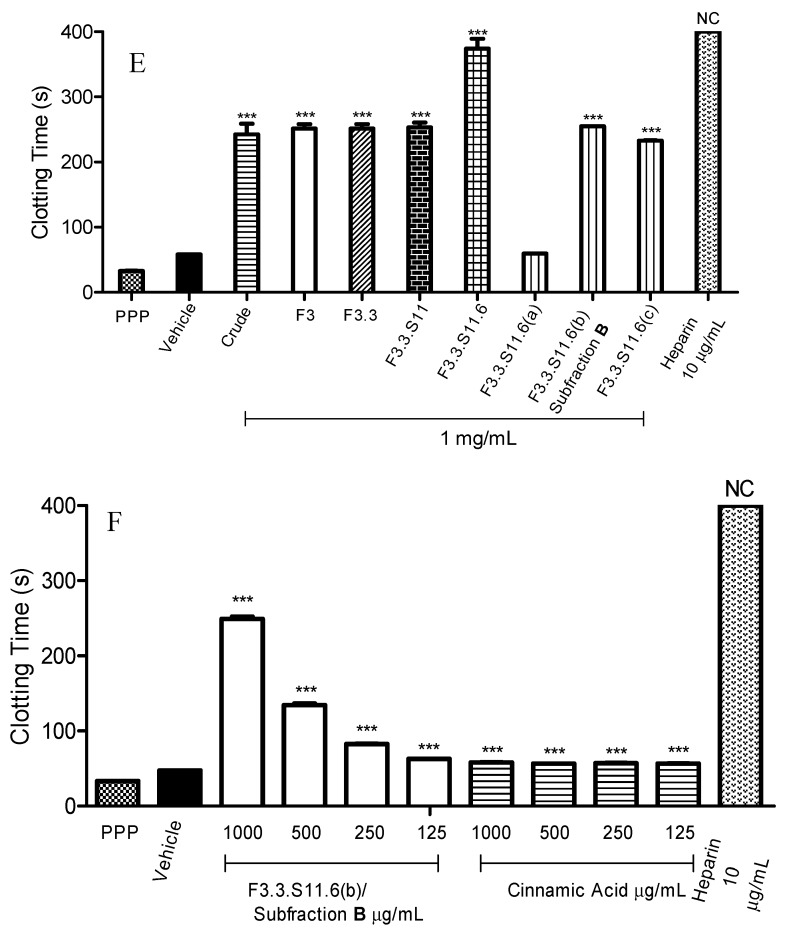
Activated partial thromboplastin time (APTT) of fractions obtained through bioassay guided fractionation. (**A**) APTT of fractions F3.1 to F3.6; (**B**) APTT of Subfraction **A**; (**C**) APTT of fractions F3.3.S11 to F3.3.S21; (**D**) APTT of fractions F3.3.S11.1 to F3.3.S11.10; (**E**) APTT of fractions F3.3.S11.6(a) to F3.3.S11.6(c); (**F**) APTT of Subfraction **B** and cinnamic acid standard. Heparin sodium salt from porcine intestinal mucosa (140 USP units mg^−1^) was used as a positive control. Data represent means ± S.E.M. of three independent experiments. Significant differences compared to control group (standard human normal pooled plasma with distilled water, vehicle) were designated as ** *p <* 0.05 and *** *p* < 0.001. NC: No Coagulation. Details of the bioassay-guided fractionation are described in Experimental Section 3.3.

Commonly, commercial anticoagulants are often associated with bleeding and other life threatening side effects. These reasons have encouraged development of alternative anticoagulants, especially from medicinal plants [24,25,26,27]. These medicinal plants were postulated to exert similar or better anticoagulant effects than commercial anticoagulants coupled with minimal side effects [16,27]. Previously, *M. malabathricum* Linn. leaf hot water crude extract was reported to show prolongation in blood clotting time through an activated partial thromboplastin time (APTT) assay, but did not prolong the blood clotting times of prothrombin time (PT) and thrombin time (TT) assays [19]. In addition, polyphenolic glycoconjugates were identified as contributors to the anticoagulant activity of *M. malabathricum* Linn. leaf hot water crude extract [20]. Other than the aforementioned information, there was no further research regarding the biological active compound(s) present in *M. malabantricum* Linn. leaf hot water crude extract that might be involved in its anticoagulant properties. In this study, in continuation of the Khoo *et al.* [20] study, the active anticoagulant solid phase extracted fraction from *M. malabathricum* Linn. leaf hot water crude extract was subjected to chromatographic separation. Among the active anticoagulant fractions F1, F2 and F3, fractions F1 and F3 were selected as candidates for further fractionation. Of the successive fractions obtained from F1 and F3, one (F3.3) possessed the most active anticoagulant activity when compared to the fractions of F1. Hence, only fractionated F3 was reported in this study. Meanwhile, F2 displayed a complex HPLC chromatogram and not well resolved peaks (data not shown). Chromatographic separations of F3 were guided by activated partial thromboplastin time (APTT) assays. Besides, the effect of the active anticoagulant fraction together with identification of potential component(s) in the intrinsic blood coagulation pathway was examined. F3, the active anticoagulant fraction with its high phenolic acids content and moderate amounts of carbohydrate and uronic acid moieties [20], was fractionated based on the separation methods for isolating polyphenolics through preparative high performance liquid chromatography (HPLC) into six fractions (F3.1–F3.6). The polyanionic characteristics of polyphenolics portray a favourable binding affinity towards blood coagulant factors [16,27]. According to Figure 1A, seemingly, all separated fractions of F3 exhibited significant anticoagulant activity except for F3.6. Subfraction F3.1 displayed the highest anticoagulant activity with a blood clotting time of 268.2 ± 7.8 s (*p* < 0.001). Meanwhile, prolongation of blood clotting times for F3.3 and F3.4 were similar. F3.3 prolonged the clotting process for about 238.1 ± 6.8 s (*p* < 0.001); while F3.4 prolonged it for about 228.8 ± 11.3 s (*p* < 0.001). The active anticoagulant fraction of F3.3 with the highest yielded was fractionated over a size exclusion Sephadex LH-20 chromatography column, and produced 21 fractions (F3.3.S1–F3.3.S21). Among these 21 fractions (F3.3.S1–F3.3.S21), ten fractions (F3.3.S1–F3.3.S10) underwent chromatographic separation and together yielded a pooled fraction designated as Subfraction A. In reference to Figure 1B, no significant difference of anticoagulant activity was observed for Subfraction A when compared to the vehicle as a negative control (*p* > 0.05). The intrinsic blood clotting times for Subfraction A and vehicle were 76.3 ± 6.6 s and 58.3 ± 2.4 s, respectively. The anticoagulant activity of Subfraction A was thus lost after the chromatographic separation.

It is presumed that the active anticoagulant compound(s) in Subfraction A, which contributed to its anticoagulant activity were eliminated. Distributions of low concentrations of the active anticoagulant compounds over two adjacent fractions might also be a cause for the observed loss of anticoagulant activities [28]. Our results paralleled those reported by Liu *et al*., [29] and Senejoux *et al*., [30] that the anti-inflammatory of *Rehmania glutinose*’s root and vasodilating properties of whole plant of *Ziziphora clinopodioides* Lam. were lost or lower than that of the crude extracts after being subjected to bioassay-guided fractionation, respectively. Both research groups suggested that the occurrence of interactions between chemical compounds, known as synergistic effects, might play a crucial role in contributing to the observed biological activities.

Owing to the fact that Subfraction A did not significantly prolong the blood clotting time, the anticoagulant activities of F3.3 fractions (F3.3.S11–F3.3.S21) were re-evaluated. Subfraction F3.3.S11 (140 mg) was found to be the most active anticoagulant fraction with a blood clotting time of 207.4 ± 12.0 s (*p* < 0.001) (Figure 1C). Apart from F3.3.S11, F3.3.S19, F3.3.S20 and F3.3.S.21 also significantly prolonged APTT times. Prolongation of blood clotting times for F3.3.S19, F3.3.S20 and F3.3.S21 were 141.1 ± 15 s (*p* < 0.001), 182.9 ± 15 s (*p* < 0.001) and 159.7 ± 14.8 s (*p* < 0.001), respectively.

**Figure 2:**
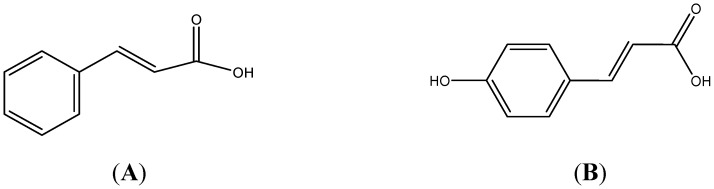
Structures of cinnamic acid (**A**) as the major component and *para*-hydroxycinnamic acid (**B**) as the minor components presented in Subfraction B.

Next, F3.3.S11, the active anticoagulant fraction, was chromatographically separated into ten fractions (F3.3.S11.1–F3.3.S11.10). Results of the APTT assay of spiked F3.3.S11 fractions are presented in Figure 1D. The data showed seven fractions (F3.3.S11.2, F3.3.S11.3, F3.3.S11.4, F3.3.S11.6, F3.3.S11.7, F3.3.S11.8 and F3.3.S11.9) that exhibited significant anticoagulant activities, with F3.3.S11.6 being the most active fraction, having 364.3 ± 4.7 s (*p* < 0.001) prolonged anticoagulation time. F3.3.S11.6 was further subjected to bioassay-guided fractionation, which yielded three fractions [F3.3.S11.6(a)–F3.3.S11.6(c)]. According to Figure 1E, F3.3.S11.6(b) also labelled as Subfraction B showed the strongest anticoagulant effect with a blood clotting time of 254.8 ± 0.3 s (*p* < 0.001). Subfraction B (a light yellow powder) was structurally characterized by ^1^H-NMR [500 MHz, D_2_O): δ (ppm): 7.99 (1H, d, *J =* 5 Hz, H-7), 7.83 (2H, d, *J =* 5 Hz, H-2, H-6), 7.72 (2H, d, *J =* 10 Hz, H-3, H-5), 7.57 (1H, d, *J =* 10 Hz, H-4), 6.85 (1H, d, *J =* 10 Hz, H-8)]; ^13^C-NMR [125 MHz, D_2_O): δ (ppm): 129.17 (C-3, C-5), 127.49 (C-4), 125.29 (C-2, C-6), 115.38 (C-8)] which suggested that Subfraction B contained cinnamic acid, and MS (EI) that revealed a mixture of two cinnamic acids: cinnamic acid itself (**A**, major component, Figure 2A) and *para-*hydroxycinnamic acid (**B**, minor component, Figure 2B).

**Figure 3:**
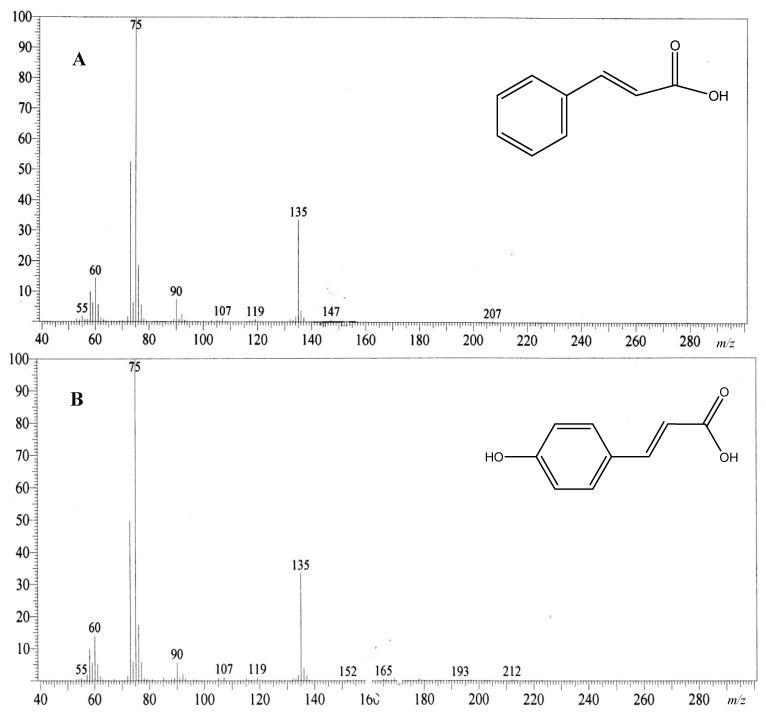
Mass spectrum of active anticoagulant Subfraction B. Subfraction B as active anticoagulant fraction was purified through a series of bioassay-guided fractionation. The anticoagulant activity was determined through prolongation of activated partial thromboplastin time (APTT). Cinnamic acid (**A**, *m/z* 147; major component) and *para*-hydroxycinnamic acid (**B**, *m/z* 165; minor component) were identified from the mass spectrum (EI).

Mass to charge ratios *m/z* (%) for cinnamic acid were 147 (M^+^, 1), 135 (34), 119 (1), 107 (1), 90 (7), 75 (100), 60 (14), 55 (2) (Figure 3A), while the mass to charge ratios *m/z* (%) for *para*-hydroxycinnamic acid was 165 (M^+^, 1), 152 (0.5), 135 (0.3), 119 (1), 107 (1), 90 (6), 75 (100), 60 (14), 55 (1) (Figure 3B). 

**Figure 4:**
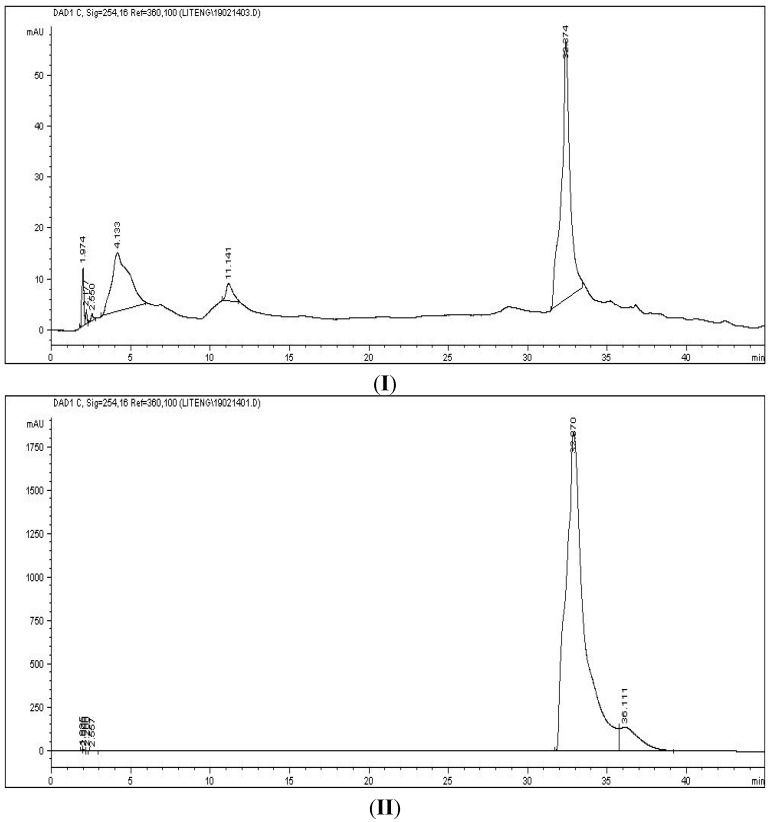
HPLC chromatograms of Subfraction B (**I**) and cinnamic acid (**II**) using a C-18 Hypersil Gold column, with water (**A**)/methanol (**B**) as mobile phase. Sample was separated under gradient elution condition at flow rate 1 mL/min. The wavelength was 254 nm.

Besides, the presence of cinnamic acid was also proved by direct comparison with a cinnamic acid standard through HPLC analysis. The HPLC profiles of Subfraction B (Figure 4I) and cinnamic acid standard (Figure 4II) matched each other. Hence, taking all the spectroscopy results into account, comparison with the HPLC profile with cinnamic acid standard (data not shown) as well as literatures data for the *m/z* values of *para*-hydroxycinnamic acid [31], cinnamic acid or the cinnamic acid derivative were suggested to act as the active ingredients in prolonging the blood clotting time in *M. malabathricum* Linn. hot water crude extract.

To verify that cinnamic acid was the active ingredient in the prolongation of APTT assay, the anticoagulant activities between Subfraction B and cinnamic acid standard were compared. According to Figure 1F, Subfraction B significantly increased the blood clotting time in a concentration dependent manner from 125 µg/mL to 1 mg/mL. Subfraction B at 1 mg/mL prolonged blood clotting to the longest time (249.1 ± 3.0 s, *p* < 0.001). At the same time, Subfraction B at 125 µg/mL showed the least potent anticoagulant effect, with a blood clotting time of 62.7 ± 0.2 s (*p* < 0.001). As shown in Figure 1F, cinnamic acid significantly prolonged the blood clotting from 1 mg/mL to 125 µg/mL (*p* < 0.001). Nonetheless, the prolonged blood clotting times for cinnamic acid were shorter compared to Subfraction B. This finding is in agreement with Kim *et al*., [32] whereby cinnamic acid standard showed mild anticoagulant activity. The blood prolongation time for cinnamic acid was 58.3 ± 0.2 s (1 mg/mL) (*p* < 0.001).

Hence, Subfraction B was suggested to contain cinnamic acid and a cinnamic acid derivative as major components in *M. malabathricum* Linn. leaf hot water crude extract. However, we postulate that the anticoagulant activity of Subfraction B might not be solely due to cinnamic acid and the cinnamic acid derivative, but might also be enhanced by other active and as yet unidentified components. Similar findings were observed by Han *et al*., who reported that *Xanthium strumarium* L. *n*-butanol fraction showed more potent anti-inflammatory and analgesic effects than isolated caffeoylquinic acid. Synergism between identified phenolic acids, which included caffeoylquinic acid, heterocyclic compounds and other unidentified secondary metabolites, enhanced the anti-inflammatory and analgesic activities for the *n*-butanol fraction [33]. Meanwhile, synergistic or additive effects for triterpene taraxasteryl myristate, taraxasteryl acetate and fern-7-en-3-β-one within *n*-hexane fraction from methanolic extract of *Scorzonera latifolia* were also observed [34]. Therefore, together with these literatures, the better blood prolongation time of Subfraction B was proposed to be due to cinnamic acid and cinnamic acid derivative, and also other different compounds.

**Figure 5:**
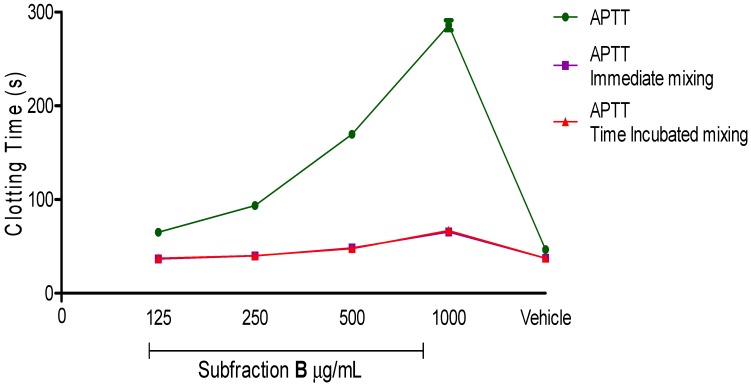
Activated partial thromboplastin time (APTT) and APTT immediate mixing as well as time incubation mixing studies of various concentrations of Subfraction B. Subfraction B as active anticoagulant fraction was purified through a series of bioassay guided fractionation. Vehicle was used as a negative control. Data represent mean ± S.E.M. of three independent experiments.

The subsequent aim of this study was to understand the effect of the active anticoagulant fraction on the intrinsic blood coagulation pathway. Subfraction B, which either acted as an inhibitor or caused coagulant factor deficiency, was determined through mixing studies. Interestingly, blood clotting times of Subfraction B mixing studies were corrected back to normal. According to Figure 5, the blood clotting time of Subfraction B was 286.0 ± 5.3 s (*p* < 0.001) at 1 mg/mL. 

After adding 50% of standard human normal pooled plasmas into spiked plasmas, the blood clotting times for immediate mixing and time of incubation mixing were corrected to 65.3 ± 1.8 s (*p* < 0.001) and 66.8 ± 0.9 s (*p* < 0.001), respectively. Both mixing studies also revealed that additional standard human normal pooled plasmas to spiked plasmas in a ratio 1:1 for concentrations at 500 µg/mL, 250 µg/mL and 125 µg/mL successfully corrected the initial blood clotting time back into the reference range (35.4–56.3 s). A plausible explanation for these results could be that Subfraction B-treated plasma exerted its anticoagulant activity through reduction of coagulant factor levels. By means of additional equal volumes of standard human normal but platelet-poor plasma in mixing studies, an insufficient amount of coagulant factors were replenished [35,36]. Hence, the blood clotting times resumed to normal, and the coagulation activity was restored.

The immediate mixing and time incubation mixing clotting times were further evaluated with the respective set of formulae, known as the Rosner index and percent correction. These interpretation methods are simple and robust to distinguish between the categories of presence of inhibitor or factor deficiency [37,38]. By using these two methods in our data interpretations, misclassification of plasma can be avoided. Based on our results, as anticipated, the Rosner indexes for immediate mixing and time incubation mixing were less than 15 (<15) (Table 1), while, their percent corrections were more than 70% (>70%) (Table 2). Both results highlighted that Subfraction B-treated plasma prolonged APTT assay abnormally due to factor deficiency. 

**Table 1:** Rosner Indexes for APTT 1:1 mixing studies of different concentrations of Subfraction B on standard human normal pooled plasmas. Subfraction B as active anticoagulant fraction was purified through a series of bioassay-guided fractionation. Values are mean ± S.E.M. (*n* = 3).

Subfraction B Concentration (μg/mL)	Immediate Mix	Time Incubation Mix
	(%)	(%)
1000	9.8 ± 0.5	10.3 ± 0.5
500	6.5 ± 0.4	6.0 ± 0.5
250	2.8 ± 0.6	3.0 ± 0.5
125	0	0.6 ± 0.3

**Table 2:** Percent corrections for APTT 1:1 mixing studies of different concentrations of Subfraction B on standard human normal pooled plasmas. Subfraction B as active anticoagulant fraction was purified through a series of bioassay-guided fractionation. Values are mean ± S.E.M. (*n* = 3).

Extract Concentration (μg/mL)	Immediate Mix	Time incubation Mix
	(%)	(%)
1000	92.2 ± 0.6	91.6 ± 0.6
500	98.6 ± 0.5	99.1 ± 0.8
250	114.1 ± 1.0	113.6 ± 0.4
125	153.3 ± 3.1	149.3 ± 1.9

Taken together, the comparison of mixing studies blood clotting times with reference range, Rosner index and percent corrections, the anticoagulant activity of Subfraction B was attributable to coagulant factor(s) insufficiency in the treated plasma. These results were further supported by a previous report, whereby *M. malabathricum* Linn. leaf hot water crude extract prolonged initial blood clotting time through reduction of intrinsic coagulant factor level(s) [19].

## 3. Experimental Section

### 3.1. Materials

Heparin sodium salt was bought from Sigma-Aldrich (St. Louis, MO, USA); while standard human pooled plasma, STA-PTT automated reagent, 0.025 M calcium chloride were purchased from Diagnostica Stago (Asnieres sur Seine, France). Methanol, acetonitrile and deuterium oxide (D_2_O) were purchased from Merck (Frankfurter Straβe, Darmstadt, Germany). Methanol and acetonitrile used were HPLC grade.

### 3.2. Plant Material, Extraction, and Solid Phase Extraction

Leaves of *Melastoma malabathricum* Linn. were obtained continuously from Lebuh Silikon, Universiti Putra Malaysia between September 2009 to December 2012. The plant material was authenticated by a botanist, Dr. Shamsul Khamis, and deposited as a voucher specimen at the Herbarium Biodiversity Unit, Institute of Biosciences, Universiti Putra Malaysia with reference number SK1717/09. The leaves were first extracted with hot water in accordance to the methodology suggested by Manicam *et al*., [19]. Briefly, cleaned and air dried fine pieces of *M. malabathricum* Linn. leaves (1 kg) were refluxed with 1 L of deionised water at 100 °C. After 5 h of extraction, a hot water extract was obtained, which was lyophilised, and subjected to solid phase extraction.

### 3.3. Bioassay-Guided Fractionation

Solid phase extraction of crude extract was carried out according to the method described by Khoo *et al*., [20]. The hot water crude extract (100 g) was separated through a 10 g C_18_, Sep-Pak Cartridge (Water, Milford, MA, USA) into four fractions (F1–F4). Active anticoagulant fractions of F1, F2 and F3 were then lyophilised and stored at −20 °C until further analyses.

The active anticoagulant fraction, F3 (8.5 g), was fractionated using an Agilent 1200 series LC system (Agilent Technologies, Santa Clara, CA, USA) preparative high performance liquid chromatography (HPLC) equipped with multi-wavelengths detector with a Zorbax Stable Bond Preparative High Throughput SB-C 18 column (21.2 × 250 mm, 7 µm). Briefly, the fraction F3 was dissolved in water: methanol (20:80, v/v) for the preparation of the final concentration of 50 mg/mL. The injection volume was 900 µL, and the mobile phase contained (A) water and (B) acetonitrile. The gradient elution profile was as follows: 0% B from 0 to 10 min, 15% B from 10 to 15 min, 20% from 15 to 30 min, 20% B from 30 to 35 min, 23% B from 35 to 40 min, 25% B from 40 to 45 min, and lastly 50% B from 45 to 50 min. The flow rate was 15 mL/min. The detection wavelength was 254 nm, and fractionated into six fractions (F3.1–F3.6). All fractions were air dried and stored in desiccators at room temperature (25 °C) prior to anticoagulant assays and consecutive fractionation to be carried out.

F3.3 (4 g) was later purified over on a size-exclusion Sephadex LH-20 column chromatography. F3.3 (400 mg) was dissolved in water: methanol (20:80, v/v) before loading onto the Sephadex LH-20 column, which was initially eluted with water: methanol (20:80, v/v) and later with water only to afford 21 fractions (F3.3.S1–F3.3.S21). These fractions were analysed and grouped accordingly to different retention factors showed on reverse phase C 18 thin layer chromatography (TLC, Merck, Darmstadt, Germany), which were eluted with water: methanol (70:30, v/v). The TLC plates were observed under UV at 254 nm and 366 nm wavelengths followed by spraying with 2 M of sulphuric acid, and heated on a hotplate at 120 °C for 5 min. Fractions (F3.3.S1–F3.3.S21) were air dried and stored in dessicator at room temperature (25 °C). Fractionation of F3.3 was repeated for a total amount of 4 g in order to achieve sufficient yield for the following fractionation steps and anticoagulant assay. Chromatographic separation of samples (F3.3.S1–F3.3.S10) and F3.3.S11 were performed on an Agilent 1100 series LC system (Agilent Technologies) equipped with multi-wavelengths detector with a Hypersil Gold^TM^ C 18 column (150 × 4.6 mm, 5 µm) (Thermo Scientific, Tewksbury, MA, USA). Each sample was prepared individually by dissolving in water: methanol (20:80, v/v) to a final concentration of 2 mg/mL. The injection volume was 20 µL and the flow rate was 1 mL/min. The mobile phase contained (A) water and (B) acetonitrile. For separation of fractions (F3.3.S1 to F3.3.S10), the gradient elution for sample separation was as follows: 0% B from 0 to 5 min, 15% B from 5 to 10 min, 20% B from 10 to 20 min, and lastly 50% from 20 to 30 min. The detection wavelengths were 210 nm and 254 nm. Chromatographic elution from 1 to 3 min of each sample (F3.3.S1–F3.3.S10) was collected and pooled together. This pooled fraction was designated as Subfraction A. Subfraction A (5 mg) was air dried and stored in a desiccator at room temperature (25 °C) for subsequent experiments. Anticoagulant activity of Subfraction A was evaluated.

Active fraction F3.3.S11 (140 mg) was separated into ten fractions (F3.3.S11.1 to F3.3.S11.10). The gradient elution profile for F3.3.S11 was as follows: 0% B from 0 to 5 min, 15% B from 5 to 20 min, 20% B from 20 to 30 min, and finally 50% B from 30 to 50 min. The detection wavelengths were 210 nm, 254 nm, 280 nm, and 360 nm. The fractions were collected based on their retention times. Five minute time slices were collected, which gave a total of 10 fractions (F3.3.S11.1–F3.3.S11.10). Each fraction was air dried and stored in desiccators at room temperature (25 °C). Fractionation of F3.3.S11 through HPLC was repeated for 140 mg in order to achieve sufficient amounts for the anticoagulant assay and further fractionations.

**Figure 6:**
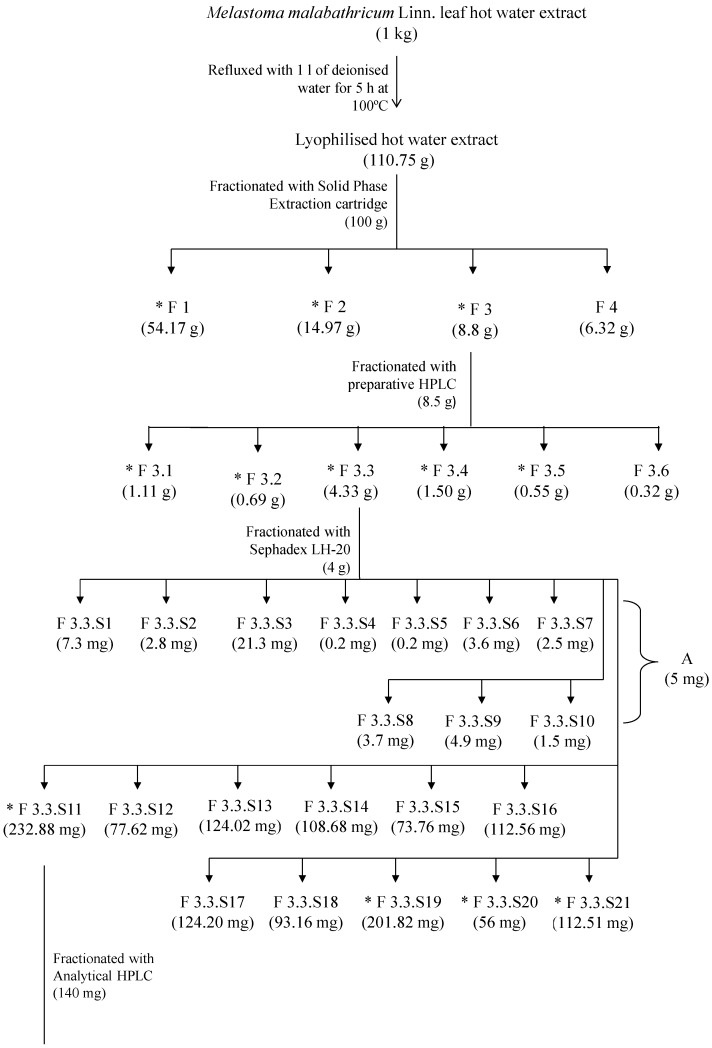

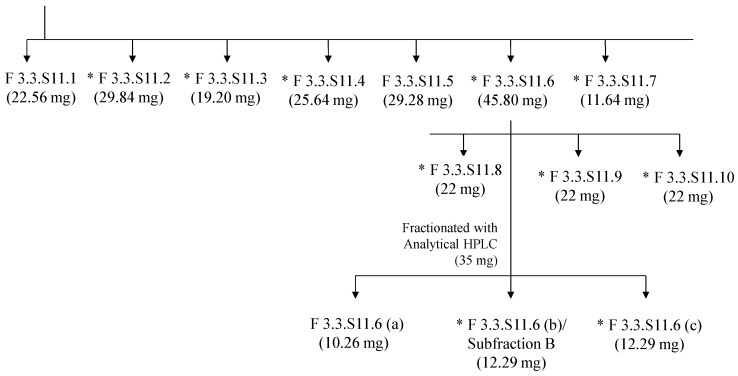
Fractionation and isolation scheme for *Melastoma malabathricum* Linn. leaf hot water crude extract.

Subsequently, the most active fraction of F3.3.S11.6 (35 mg) was separated on a Jasco 1500 series LC system equipped with a refractive index detector (Jasco international, Hachioji, Tokyo, Japan) with a Rezex^TM^ RCM-Monosaccharide Ca^+2^ column (300 × 7.80 mm, 8 µm) (Phenomenex, Torrance, CA, USA). F3.3.S11.6 was prepared with a final concentration of 10 mg/mL by dissolving with deionised water. The injection volume was 20 µL/min. The mobile phase contained water, and was separated by isocratic elution. The flow rate was 0.6 mL/min. Fractionation of F3.3.S11.6 was based on refractive index intensity, which gave three fractions [F3.3.S11.6(a), F3.3.S11.6(b) and- F3.3.S11.6(c)]. F3.3.S11.6(b) was also designated as Subfraction B. Fractionation of F3.3.S11.6 was repeated until sufficient amounts (12.29 mg) of fractions [F3.3.S11.6(a), F3.3.S11.6(b), F3.3.S11.6(c)] were obtained. Fractions collected at the same retention times were combined together and lyophilised. Each lyophilised sample was stored in a desiccator at room temperature (25 °C). The anticoagulant activities of fractions [F3.3.S11.6(b)–F3.3.S11.6(c)] were subsequently examined. The most active Subfraction B was subjected to spectroscopic and high performance liquid chromatography analyses in order to elucidate its active components. The fractionation and isolation scheme for *Melastoma malabathricum* Linn. leaf hot water crude extract is shown in Figure 6.

### 3.4. Analytical Identification of Active Principle

The mass spectrum of active anticoagulant Subfraction B was recorded on a Shimadzu gas chromatograph-mass spectrometer (GCMS-QP5050A, Shimadzu, Nakagyo-ku, Kyoto, Japan) coupled with a direct insertion probe (DI). Analysis using this mass spectrometer was performed using 70 eV electrospray ionisation (EI) in the positive mode.

* Fractions or compounds with anticoagulant activity.

The nuclear magnetic resonance (NMR) spectra (^1^H and ^13^C) were recorded on a Varian INOVA NMR spectrometer (Agilent Technologies, Santa Clara, CA, USA). This NMR was operated at 500 MHz for ^1^H and 125 MHz for ^13^C, and controlled by the Vnmrj software. Subfraction B was analysed by dissolving in deuterium oxide (D_2_O). The chemical shifts (δ) were expressed in ppm (parts per million), while coupling constants (*J*) are reported as Hz. Multiplicities of proton signals were described as singlet (s) and doublet (d). All NMR spectra were analysed using offline ACDLabs 12.0 software (Advanced Chemistry Development, Toronto, ON, Canada).

Subfraction B was analysed using an Agilent 1100 series LC system (Agilent Technologies) with a Hypersil Gold^TM^ C 18 column (150 × 4.6 mm, 5 µm) (Thermo Scientific). Subfraction B and cinnamic acid standard were dissolved in water: methanol (20:80, v/v) for preparation of the final concentration at 1 mg/mL. The injection volume was 20 µL. The mobile phase contained (A) water: formic acid (99.9:0.1, v/v) and (B) acetonitrile. The gradient elution profile was as follows: 0% B from 0 to 5 min, 5% B from 10 to 15 min, 10% B from 15 to 20 min, 20% B from 20 to 30 min, and lastly 50% B from 30 to 45 min. The detection wavelength was 254 nm.

### 3.5. Anticoagulant Assay

Anticoagulant activities of all fractions, subfractions, cinnamic acid, and mixed plasmas were evaluated using activated partial thromboplastin time (APTT) assays. Fractions, subfractions and cinnamic acid were dissolved in distilled water for preparation of the final concentration of 1 mg/mL. Samples were spiked with reconstituted standard human pooled plasma in a ratio 1:1. Spiked samples were later added with reconstituted STA-PTT automated reagent and 0.025 M of calcium chloride. Blood clotting time was recorded until the formation of blood clot was observed. These APTT assays were recorded using a STA Compact coagulation analyser (Diagnostica Stago, Asnieres sur Seine, France). Spiked heparin sodium salt (10 mg/mL) and spiked distilled water (vehicle) were used as positive and negative controls, respectively.

### 3.6. Mixing Studies

Mixing study is a testing guideline used to distinguish presence of inhibitor or factor deficiency for causing abnormal prolongation in activated partial thromboplastin time (APTT) assay [35,37]. In this study, two types of mixing studies, immediate mixing study and time incubation mixing study, were performed.

#### 3.6.1. Immediate Mixing Study

Different concentrations of Subfraction B (1 mg/mL, 500 µg/mL, 250 µg/mL and 125 µg/mL) were spiked with equal volume of standard human normal pooled plasmas. Next, spiked samples were mixed again with standard human normal pooled plasma in a 1:1 ratio, and designated as mixed plasmas. Immediately, the mixed plasmas were subjected to APTT analyses.

#### 3.6.2. Time Incubation Mixing Study

Subfraction **B** with different concentrations (1 mg/mL, 500 µg/mL, 250 µg/mL and 125 µg/mL) were spiked with standard human normal pooled plasmas. Spiked samples and standard human normal pooled plasmas were incubated separately at 37 °C water bath for 2 h in air tight tubes. After that, both incubated spiked samples and standard human normal pooled plasma were mixed in a 1:1 ratio, and identified as mixed plasmas. APTT assays for time incubation mixed plasmas were determined by a STA Compact coagulation analyser (Diagnostica Stago).

#### 3.6.3. Rosner Index

Rosner index was calculated for all 1:1 mixed plasmas results from the immediate and time incubation mixing studies with modified formula. This is an index of sample correction with a prolonged APTT profile. The formula of this index is as follows:

$$\text{Index}=\;\frac{\text{APTT }1:1\text{ mix}-\text{APTT of vehicle}}{\text{APTT of SS}}\;\times 100$$

where SS = Spiked sample

An index of correction of less than 15 (<15) suggests a factor deficiency present in the mixed plasma, whereas an index of correction of more than 15 (>15) suggests the presence of an inhibitor in mixed plasma [38].

#### 3.6.4. Percent Correction

Modified percent correction from Chang *et al*., was used to interpret the mixing study results. Blood clotting effects of spiked samples after mixed with standard human normal pooled plasma in a ratio (1:1) were evaluated. The formula of percent correction was displayed as follows:

$$\text{Percent Correction}=\;\frac{\text{APTT of SS}-\text{APTT }1:1\text{ mix }}{\text{APTT SS}-\text{APTT of Vehicle}}\;\times 100\text{\%}$$

where SS = Spiked sample

Percent correction of more than 70% indicates the presence of a factor deficiency, and causes prolongation of the APTT assay. A low percent correction of less than 58% indicates the presence of an inhibitor. A percent correction between 70%–58% is classified as an undefined result [39].

### 3.7. Statistical Analysis

Data shown were expressed as mean ± S.E.M. in triplicates. Significance differences for all tests were referred as * *p* < 0.05, ** *p* < 0.01 and *** *p* < 0.001. All data except Rosner index and percent correction formula were analysed using one way analysis of variance (ANOVA) followed by *post-hoc* Dunett’s multiple range tests, while the Rosner index and percent correction formula were analysed by student’s t-test (GraphPad Prism v.5.0, La Jolla, CA, USA).

## 4. Conclusions

In conclusion, this is the first report to show that cinnamic acid and a cinnamic acid derivative identified in *M. malabathricum* Linn. leaf hot water crude extract exhibited anticoagulant activities. Bioassay-guided fractionation of *M. malabathricum* Linn. leaf hot water crude extract suggested that cinnamic acid and the cinnamic acid derivative appeared to be the major compounds in Subfraction B, although the anticoagulant activity was not totally dependent on cinnamic acid and the cinnamic acid derivative. The presence of other unidentified compounds in Subfraction B could also be responsible for the prolonged blood clotting time. Moreover, Subfraction B normalised blood clotting times in both the immediate mixing and time incubation mixing studies. Subfraction B prolonged the blood clotting time by causing a factor deficiency in the intrinsic pathway. Therefore, the underlying intrinsic blood coagulation pathway affected by Subfraction B remains to be further examined in future studies. This study may provide new insights as Subfraction B may be a potential candidate in developing a safe and natural anticoagulant for preventing and treating thromboembolism.
